# Evaluation of Greek psychiatric reforms: methodological issues

**DOI:** 10.1186/1752-4458-7-11

**Published:** 2013-03-28

**Authors:** Evangelia Loukidou, Anastasios Mastroyannakis, Tracey Power, Graham Thornicroft, Tom Craig, Nick Bouras

**Affiliations:** 1 C.M.T. Prooptiki, Athens, Greece; 2 Maudsley International, King’s College London, Institute of Psychiatry, De Crespigny Park, London, SE5 8AF, UK; 3 King’s College London, Institute of Psychiatry, England, UK; 4 Health Service and Population Research Department, King’s College London, Institute of Psychiatry, England, UK

**Keywords:** Evaluation, Mental health reforms, Community mental health services, Greece

## Abstract

Over the last three decades significant efforts have been made in many European countries to move away from a mental health system dominated by institutional care towards one whereby the main emphasis is on providing care and support within the community. Although the time of starting the reforms, their pace, the political context, and the exact objectives varies substantially across Europe, practically all countries have been undergoing such major reforms aimed at establishing services in the community to replace institutional based care. Each country makes its own decisions about the necessary mental health services taking into account a range of factors including population needs, level of resources, flexibility and coordination of organizational structures, as well as local culture. These factors become an integral element of a national mental health policy and action plan, closely linked with national public health strategies.

Greece has been modernizing an outdated mental health system, which was based on institutional care, over the last 20 years, by developing community-based mental health care. This article describes the methodology used for the evaluation of the Psychargos programme of the mental health reforms in Greece. Various forms of community-based mental health services have been developed including supported living facilities, community mental health centres and employment opportunities.

## Introduction

Over the last three decades significant efforts have been made in many European countries to move away from a mental health system dominated by institutional care towards one whereby the main emphasis is on providing care and support within the community [1] (WHO, 2011). Although the time of starting the reforms, their pace, the political context, and the exact objectives varies substantially across Europe, practically all countries have been undergoing major reforms aimed at establishing services in the community to replace institutional based care.

Each country makes its own decisions about the necessary mental health services taking into account a range of factors including population needs, level of resources, flexibility and coordination of organizational structures, as well as local culture. These factors become an integral element of a national mental health policy and action plan, closely linked with national public health strategies.

Various forms of community-based mental health services have been developed including supported living facilities, community mental health centres and employment opportunities. The evidence supports the validity of developing a “balanced care approach” between community and hospital services in all countries regardless of resources [2,3].

Developing these services and providing sufficient resources to sustain them is critical, as is effective coordination with other sectors, such as social care, housing and employment, and collaboration with service users and family groups. The needs of the mental health workforce should also not be overlooked when considering the balance of services. A well-trained workforce is a prerequisite for quality services. Training should not be restricted to mental health-related skills alone. There is also a need for training in organizational and managerial skills, which are lacking particularly in some countries, hampering reform and the coordination of multi-agency services.

It is also necessary to put in place systems that help to strengthen the evidence base about what works and in what context. We still have little information about either the effectiveness or the cost–effectiveness of many interventions and methods of delivering services. In addition we are lacking established methodology for evaluating the development, organization and function of mental health systems as a whole.

Over the last 20 years, Greece has been modernizing an outdated mental health system, which was based on institutional care. The implementation of an extensive transformation became possible through the “Psychargos” programme, a national strategic and operational plan, developed by the Greek Ministry of Health and Social Solidarity. The last phase and most comprehensive of the Psychargos programme lasted from 2000 to 2010 and was jointly funded at 75% of the cost by the European Union and 25% by the Greek State over a period of 5 years, after which the entire cost of the new services became the responsibility of the Greek National Budget. The Psychargos programme became almost synonymous with the psychiatric reforms in Greece including the deinstitutionalization of chronic psychiatric hospital patients and the development of a wide range of community mental health service.

A retrospective evaluation, known in the management terminology as “ex-post evaluation”, of the implementation of the “National Action Plan Psychargos 2000-2010” of the psychiatric reforms was commissioned at the end of 2010 by the Greek Ministry of Health at the request of the European Union. The evaluation team consisted of independent assessors from abroad who had not been involved with the planning and the implementation of the Psychargos programme and who were assisted by an expert Greek team. This paper presents the methodology used for the evaluation.

## Methodology

The basis of the evaluation stemmed from the European Guide [4] that defines evaluation as “the systematic and objective assessment of an on-going or completed project, programme or policy, its design, implementation and results. The aim is to determine the relevance and fulfilment of objectives, development efficiency, effectiveness, impact and sustainability”.

The essential issues that need to be addressed in any evaluation are

• The intervention strategy, including the rationale behind the Psychargos programme and more specifically, why certain priorities were selected in relation to geographical areas, population needs, existing problems, advantages of certain interventions against other alternatives.

• The selection of specific evaluation questions emerged through an analysis of implemented interventions, requirements of the commissioners of the services, and views of key stakeholders concerning issues that warrant investigation.

The methodological approach of the present project adopted quantitative and qualitative approaches based on multiple research methods through diverse sources and participants [5]. The incorporation of the qualitative dimension broadened the evaluation’s scope to aspects such as organization, operation, co-ordination of the service system and the impact of changes to personnel and service users.

Quantitative measures of the evaluation included indicators of the implementation of the project’s diverse actions (percentages of completed actions, number of deinstitutionalized patients, percentages of staff who had attended training projects related to deinstitutionalization).

Qualitative indicators included the aims and policies of the country’s mental health care, the success of efforts in achieving the psychiatric reforms and the identification and adoption of quality standards set by relevant stakeholders [6]. Incorporating this qualitative dimension required restricting the term “psychiatric reform” to specific categories according to:

• The strategic objectives of the reform

• Issues that have emerged from past evaluations

• International guidelines regarding systems’ evaluation

Based on the above applied methodological considerations, data were collected from primary and secondary data sources.

Primary Data Sources included:

• Individual interviews

• Focus groups

• Site visits

• Questionnaires

Secondary Data Sources included:

• Literature reviews

• Reports

• Official Documents

### Data collection

The methods and the process that were employed for gathering the data were the following:

### Planning meeting

During the preparatory stage of the project, members of the evaluation team carried out an introductory meeting with the stakeholders of the Psychargos programme, including Governmental officials from the responsible Ministries and other relevant organizations. The outcome of this initial meeting was that the evaluation should not be limited to the “Psychargos” programme but should involve the broader system of mental health services as the mental health reforms in Greece extended over a longer period than that covered by the Psychargos programme. As there was lack of official monitoring and financial data, information was gathered concerning the number and type of services that were operational, to what extent the programme’s targets had been achieved and how were the quality, monitoring and evaluation procedures ensured.

### Individual interviews

Individual interviews provide detailed information about the function of different structures of the service system. A semi-structured interview was devised, which covered the following areas: objectives, achievements of the Psychargos programme, quality improvement of mental health services, liaison and co-ordination of services, staff training, positive and negative aspects of the reformed mental health system, and proposals for future actions. Individual interviews were conducted face to face with the interviewee by a member of the evaluation team, who led the discussion based on the semi-structured questionnaire. Members of the evaluation team met approximately 150 mental health mostly senior clinicians, clinical academics and managers from the public and NGOs sectors. The duration of the interviews ranged from one (the shortest) to three hours. Individual interviews were also carried out with a small number of service users met on the onsite visits in visited providences.

### Focus groups

Two separate focus groups were convened, one with service providers from diverse backgrounds and expertise and a second one with service users and families. The aim of the focus groups was to collect information on the overall organization, operation, management and effectiveness of the mental health system. The focus groups captured the broad views of participants concerning the strengths of the programme and identified key issues for further exploration.

Both focus groups dealt with the overall operation and effectiveness of the mental health system as well as with the process of the psychiatric reforms. Communication was supported by an interpretation service.

The service providers’ focus group consisted of 30 people, most of whom came from diverse mental health disciplines (psychiatry, child psychiatry, psychology, social work, including some managers) and represented the broad spectrum of mental health services (public sector, NGOs, university departments, scientific committees, special committees, etc.). During the five hour procedure, participants were asked to present their views on a list of subjects that the evaluation team had prepared. The second focus group consisted of 15 service users and users’ families and lasted 2 hours. The same process was followed for this group as with the first one.

For the selection of service providers’ focus group, attention was given to the representativeness of participants according to the following criteria:

• Degree of responsibility in the planning and implementation of the Psychargos programme.

• Professional background and practical knowledge related to the provision of mental health services that were developed through the Psychargos programme.

• Category of service provided (Psychiatric hospitals, Community Mental Health Centres, Outreach Services (known as Mobile Units) and legal status (Public sector, NGOs, voluntary organizations) of the mental health service that participants were representing.

• Geographical distribution of the participating services.

Sampling for the users’ focus group was formed through convenience selection of members of organizations developed by users and users’ families throughout Greece. Attempts to include users who did not belong to such organizations were not successful.

### Observation site visits

Site visits help making personal contacts and provide an opportunity to verify specific parts of the data collection. Site visits create also a climate of trust between the evaluators and the respondents.

Site visits took place in a range of different mental health units in order to form a direct view and opinion relating to their physical and operational infrastructure. During these visits additional data were collected with individual interviews with members of front line staff and service users and review of local policies and documents increasing and strengthening the representativeness of the collected data. During the site visits, the evaluation team interviewed multi-disciplinary mental health professionals and front line staff including psychiatrists, psychologists, nurses, social workers, therapists, clinical directors, managers and also service users. Mental health services were visited in Athens and in additional four provincial cities and towns. The services visited covered a wide range of mental health services including residential supported units, mental health community centres, social enterprises-KOISPE, day centres, psychiatric hospitals and psychiatric units in general hospitals.

### Questionnaires

Specially devised self-administered questionnaires were used in order to triangulate data on quality indicators such as organization, operation and management of mental health services including care environment, staff perceptions and skills, applied treatments and respect for service users’. Questionnaires were distributed electronically to a broad range of mental health services, including the public sector and non-governmental organisations (NGO). The questionnaires were completed by the lead professional or manager of the randomly selected services. The sample consisted of 16 NGOs and 8 public sector providers. In total 100 questionnaires were distributed covering the quality indicators referred above. Ten NGOs and 3 public providers responded and 49 questionnaires were returned.

### Documentation and literature review

The collection of data was supplemented by reviewing a large number of official documents and reports. These documents were related to the design, planning and organization of services, they outlined targets, expected outcomes of the psychiatric reforms in general and in particular the implementation of the Psychargos programme. In addition documents were reviewed relating to the financial cost of the development and the operation of the new services with the Psychargos programme. Existing data were also reviewed in relation to the numbers deinstitutionalized population, hospital admissions and re-admissions rates and average length of in patient.

A literature search was also carried out on the international experience of deinstitutionalisation and also on publications referring to Greek psychiatric reforms e.g. [7-15]. There were also several studies published in Greek that were reviewed.

## Results

One of the great achievements of the Psychargos programme was the closure of 5 psychiatric hospitals. The remaining 3 are due to close by 2015. In respect to the introduction of psychiatric services in general hospitals, the aim was partially met, as only 30 of the planned 75 psychiatric units were opened. However, an additional 9 hospitals were expected to provide mental health services, according to data provided by the Governmental Mental Health Directorate.

There were over 60 NGOs providing mostly residential care, day care and outreach teams known in Greece as “mobile units”. We noticed that several of the NGOs had limited capacity providing 1–2 services either residential or day care.

The highest rates of target achievement were: sheltered apartments 211% of the planned number. These apartments may be flats or houses and accepted people with psychiatric disorders or intellectual disabilities and mental health problems requiring support for developing their self-care skills. The maximum number of residents in a sheltered apartment was 6 people and staff was peripatetic visiting them at regular times. Alzheimer’s centres reached 180% of the target. Day centres for people with a wide range of mental health problems reached 95%. Boarding houses reached 89%. These were community residential places mostly purposed built for up to 25 patients with 24 hours high staff support for mostly old age adults and people with intellectual disabilities. Outreach teams known as mobile units in Greece and operating in the islands and mostly remote areas reached 68% of the target. Guest houses accommodated up to 15 people with chronic mental illness and psychosocial problems with 24 hours staff support in several purpose built places reached 52% of the target.

The development of community mental health centres was partially achieved 43% of the target and social enterprises, providing employment reached only 33%.

Table 1 summarises the programmed and actually developed mental health units and the success rates for each of them.

**Table 1:** Planned and actually developed mental health units

**Mental health units**	**2001 (baseline)**	**2010 (target)**	**2010 (achievement)**	**Target achievement 2001-2010**	
				**Number**	**%**
Psychiatric Hospitals*	9	4	3	−1	**125**
Psychiatric & Child-Psychiatric Units of General Hospitals		75	41	−34	**55**
Psychiatric Units of General Hospitals			30		
Child-Psychiatric Units of General Hospitals**			11		
Mental Health Centers	28	80	34	−46	**43**
Mental Health Centers for children ***	22	73	10		
Mobile Units	6	40	27	−13	**68**
Day Centers	18	42	40	−2	**95**
Psychosocial Rehabilitation Units	196	407	430	23	**106**
Guest Houses	95	170	88	−82	**52**
Boarding Houses	16	130	116	−14	**89**
Sheltered Apartments	85	107	226	121	**211**
Socio-vocational rehabilitation units	102	148	102	−46	**69**
Alzheimer’s Centers		5	9	4	**180**
Drug abuse Centers		35	0	−35	**0,00**
Alcohol abuse Centers		15	0	−15	**0,00**
Social Enterprises -KOISPE		55	18	−37	**33**
Home Care Unit		✓	1	1	
Autism Center for children		✓	2	2	

*Includes Psychiatric Hospitals in full operation. Additionally the University Psychiatric Hospital “Aiginition” operates but does not have long-stay units.

** It is not reported in the First Revision of Psychargos (Ministry of Health & Social Solidarity 2001) neither the baseline number in 2001 nor the target-number for the development of Child-Psychiatric Units in General Hospitals. Therefore the success rate cannot be deduced.

***According to the First Revision of Psychargos (Ministry of Health & Social Solidarity 2001) the mental health centers for children were 22 in 2001. However, according to data provided by the Mental Health Directorate, in 2010 there were only 10 centers (mental health centers for adults that also provide services for children have not been included). Therefore the success rate cannot be deduced.

The Psychargos programme included a number of specialized training programmes that were carried out for all staff employed in the newly-developed units. These programmes included theory and practice and educational visits to similar services in other countries. Several thousand employees attended these programmes that reached 97% of the initial set target.

Targets related to the development of Social Enterprises for Employment were not achieved at satisfactory levels. With the exception of the regions of Attica, around Athens, (78%) and Ionian Islands (100%), the rates in the other regions were low and in some regions there no Social Enterprises for Employment.

An important factor in assessing the quality of a system is to collect information on the views and perspective of those managing and working in the system and those who ultimately use it. The focus groups for mental health professionals and for service users and their families were also revealing in their similarities and differences as described by Loukidou et al. [16]. Focus groups have been used in qualitative mental health research. Focus groups allow people to build on others’ responses and come up with ideas they might not have thought of in a one on one interview [17]. They are very cost effective in terms of gathering primary data and are very time efficient.

The observation site visits were also very useful as they allowed direct contact with developed services, professionals and service users. The visited services were chosen to be representative of the overall sample of the country’s mental health services. One of the findings from the site visits was the discrepancy between the physical infrastructure of several community settings and the provided services. There were some buildings of high standard which appeared to have more capacity than the services they provided, which in some cases were mostly restricted to providing forms of psychotherapy resembling out patient clinics. In contrast, some settings seemed over-provided with professionals for the nature of the client population. So for example there were a number of residential care settings that were very highly staffed with qualified professionals (psychiatrists, psychologists, therapists, social workers, etc.) attending on a regular daily or weekly basis but mainly providing ‘ supportive’ care with little emphasis on progression giving a distinctly institutional flavour of care. This type of provision can prevent service users from progressing towards more independent living which is one of the main goals of community mental health. Some residential facilities had been set up far away from the hospital to which they would be admitted should they relapse and with little apparent consideration given to the location of other mental health facilities or family support.

The psychiatric units visited in general hospitals showed a different picture to community based services. Their physical infrastructure was rather outdated and unkempt and all commented on their limited capacity to meet local needs. The number of staff (particularly nursing) was not adequate. In all sites there was a mixture of patients’ gender, age ranging from young adults to elderly people with a variety of diagnoses from psychotic illness, depression to personality disorders.

The questionnaires provided some data on the requested information of quality indicators but this was not as enlightening as expected. The response rate was 49% but there were several unanswered questions preventing a meaningful analysis.

The main strengths of the mental health reforms as identified by the current evaluation were: (a) an overall substantial service transformation towards developing modern community based mental health services focussed on deinstitutionalisation with extensive reduction of hospital-based long stay accommodation including the entire closure of some psychiatric hospitals; (b) a wide range of community services in several parts of the country including Community Mental Health Centres (CMHC), different types of residential provision, day centres and hospitals, outreach/mobile mental health units and vocational services’; (c) reports that local communities were becoming gradually more accepting of people with mental illness; (d) illustrations of positive changes in the attitudes of staff towards more person-centred care and examples of notable individual leadership; (e) a few examples of active social firms or co-operatives offering sheltered work; (f) examples of mental health promotion activities aiming to raise community and general public awareness by CMHCs, NGOs and other organisations. In addition there was an active anti-stigma campaign linked with similar international programmes, with indications of real progress in reducing stigmatisation.

The main weaknesses of the reforms included: (a) an overall impression of patchy, ill-coordinated and often inadequate provision on the ground with weak implementation of agreed policies; (b) a lack of a population-based approach to the mental health system, without clear evidence for assessing the needs of local populations and no clear understanding at the local level of what components are necessary for a comprehensive system of care; (c) inequity in the development of services between different areas around the country; (d) important services gaps were noticed for child and adolescent mental health services, services for older adults and specialist services for people with autistic spectrum disorders, those with intellectual disabilities, eating disorders and forensic psychiatric services; (e) no quality assurance mechanisms and systems for clinical governance; (f) service users’ involvement and carer advocacy remained underdeveloped despite some progress and the fact that there are some organisations in place.

## Discussion

Although evaluations of certain components of mental health care have been reported already, including implementation of policy [18-21], evaluation of an entire National mental health system is rare.

Many of the planned actions of Psychargos programme were successfully implemented in reforming the mental health system in Greece. There were, however, inequalities between urban and rural areas and several of the targets showed considerable variation. For instance, establishing psychiatric departments within general hospitals constituted one of the main targets of the psychiatric reforms and was achieved in a few regions but their rate of implementation varied widely across the country. A similar situation of important variation was present for the development of community mental health centres across the country.

The development of community services was uneven, as the focus was placed largely on the establishment of residential services in order for the deinstitutionalization of chronic psychiatric hospital patients, which in some cases over-achieved the initial target. In some cases, targets had not been achieved at all (crisis management centres for drug and alcohol abuse).

In the course of the project the evaluation team came across several limitations in collecting the required data. Most of the problems were related to the lack of statistics and monitoring data. This was particularly obvious with financial data relating to expenditure and cost of most of the developments. The only available data were the annual budgets of NGOs but without breakdowns of staff or unit cost or activity data. That meant it was not possible to carry out any cost benefit analysis or any other form of economic evaluation. There was also a lack of epidemiological and local population needs data, financial data for public services, and also data on the number and distribution of personnel. In addition there was lack of a unified and updated monitoring database regarding the operating services and the activities carried out by them. This serious deficiency of data did not allow for comparisons to be made between the different parts of the services. The shortage of monitoring mechanisms as well as the application of specific quality standards regarding the operation of services prohibited the evaluation of the system according to clearly defined criteria and standard procedures.

Nevertheless with the qualitative methods used in this evaluation, namely focus groups for service providers and users’ as well observations from site visits, we were able to cross-validate our data with existing documents concerning the design, planning and implementation of the Psychargos programme.

The evaluation report highlighted the risks posed by the weakness of the reforms unless remedial actions took place. In May 2012 the Greek Ministry of Health published a new plan the Psychargos C programme for continuing the mental health care reforms having taken into consideration some of the recommendations of the evaluation described in this article. However, the current very difficult financial situation in Greece presents a serious problem for the continuation of the psychiatric reforms and there is a risk for the achieved improvements to be at least partially reversed.

Problems with coordination of services have also been described with psychiatric reforms in other European countries [18,19,22,23]. In Norway during the ten year National Plan for Mental Health, there was significant growth and development of primary care in relation to mental health. Almost all the targets for increased resources were achieved, but there was uncertainty regarding the quality of care improvement [24]. In Australia and New Zealand the development of community oriented mental health services reported to have been ‘a long term challenge’ McGeorge [25].

In a comprehensive study of several European countries Senbrau et al. [20] reported that while a few countries have been successful in the implementation of community based mental health services, in many others access to community based services is still very limited and may commonly consist of small pilot projects [26,27].

In today’s complex landscape of services for people with mental health problems the number of possible interfaces between services is increasing. Together with existing fragmented financing systems, these interfaces are increasingly difficult to manage in terms of providing optimal care pathways adjusted for diagnosis, disability, and needs for the service user and his/her family [28].

A study by the European Union and the World Health Organisation compared mental health care systems across Europe [29]. The report’s conclusion that the best policies and practices could be found in the English National Health Service (NHS) based on data provided by central government departments, although outcomes or cost-effectiveness were not assessed. England has among the highest rates of mental health and other social problems in Europe [30], and spends a larger proportion of its health budget on mental health than any other country in the continent [31]. At the time of the EU/WHO study there had been significant increase in spending on mental health services. While the report highlighted the strengths of the English system, some psychiatrists have expressed concerns about the actual quality of assessment and care that patients receive [32].

Many countries have good mental health strategies, proposing comprehensive models of community-based mental health services, but they are struggling to implement them. Such strategies require major changes in service structures and ways of working, additional investments in services, staff numbers and training and the commitment of many agencies [33].

The presented methodology of reviewing and evaluating a mental health system at a National level, as the evaluation of the Psychargos programme, offers certain advantages. These include the incorporation of quantitative and qualitative dimensions which broadened the evaluation’s scope to aspects such as organization, operation, co-ordination of service system and the impact of changes to personnel and service users.

There are, however, limitations in the described method which can be summarized as containing a degree of subjectivity unless rigorous sampling is applied. This was not possible in the current project mostly because of the serious limitations of the available official data. Another limitation is that the private sector providers in Greece, representing a considerable proportion though the size is not available from official statistics, were not included in the specifications of the evaluation. Until a more advanced methodology of mental health systems evaluation is embraced, the methodology used for the Psychargos programme can be considered for further evaluations of National mental health care programmes.

## Competing interests

The authors declare that they have no competing interests.

## Authors’ contributions

EL & TM carried out the collection of data and contributed in drafting the manuscript. TP, TC, GT and NB contributed to the design of the study and in drafting the manuscript. All authors read and approved the final manuscript.
